# Supplementary material for: Antihypertensive Activity of the Alkaloid Aspidocarpine in Normotensive Wistar Rats
Source: Molecules. 2022 Oct 14;27(20):6895. doi: 10.3390/molecules27206895 (PMC9609921; doi:10.3390/molecules27206895)
Supplement: Supplementary file 1 [file molecules-27-06895-s001.zip › molecules-1936979-supplementary.pdf]

Supplementary Materials

Antihypertensive Activity of the Alkaloid Aspidocarpine in Normotensive Wistar Rats

Noemi Oliveira Monteiro[1], Theresa de Moura Monteiro[3], Thalya Soares R. Nogueira[2], Jacqueline Rodrigues Cesar[2], Lara Pessanha S. Nascimento[2], Karoline Azerêdo Campelo[2], Graziela Rangel Silveira[2], Fernanda Antunes[3], Daniela Barros de Oliveira[1], Almir de Carvalho Junior[4], Raimundo Braz-Filho[2,5] and Ivo José Curcino Vieira[1,2*]

[1]Laboratório de Tecnologia de Alimentos, CCTA, Universidade Estadual do Norte Fluminense Darcy Ribeiro, Campos dos Goytacazes 28013-602, Brazil

[2]Laboratório de Ciências Químicas, CCT, Universidade Estadual do Norte Fluminense Darcy Ribeiro, Campos dos Goytacazes 28013-602, Brazil

[3]Laboratório de Clínica e Cirurgia Animal, CCTA, Universidade Estadual do Norte Fluminense Darcy Ribeiro, Campos dos Goytacazes 28013-602, Brazil

[4]Instituto Federal de Educação, Ciência e Tecnologia da Bahia, campus Vitória da Conquista, 45078-300, Brazil

[5]Departamento de Química Orgânica, Instituto de Química, Universidade Federal Rural do Rio de Janeiro, Seropédica, Rio de Janeiro 20000-000

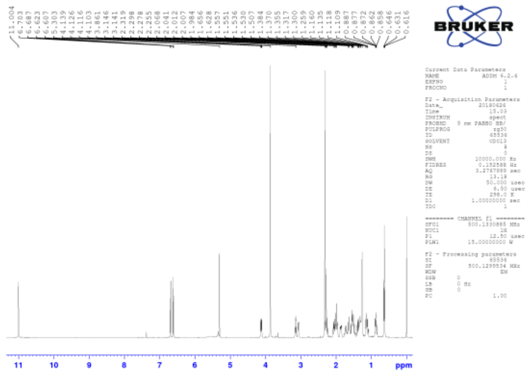

Figure S1: ^1H NMR spectrum of Aspidocarpine ($CDCl_3$, 500 MHz).

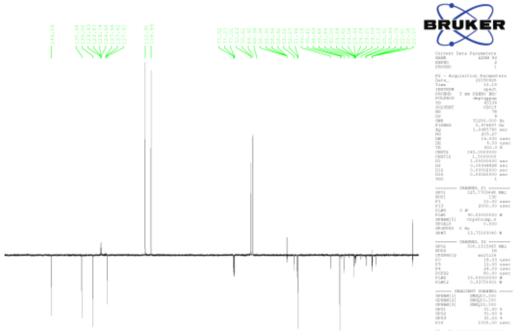

Figure S2: ^{13}C DEPTQ NMR spectrum of Aspidocarpine ($CDCl_3$, 125 MHz).

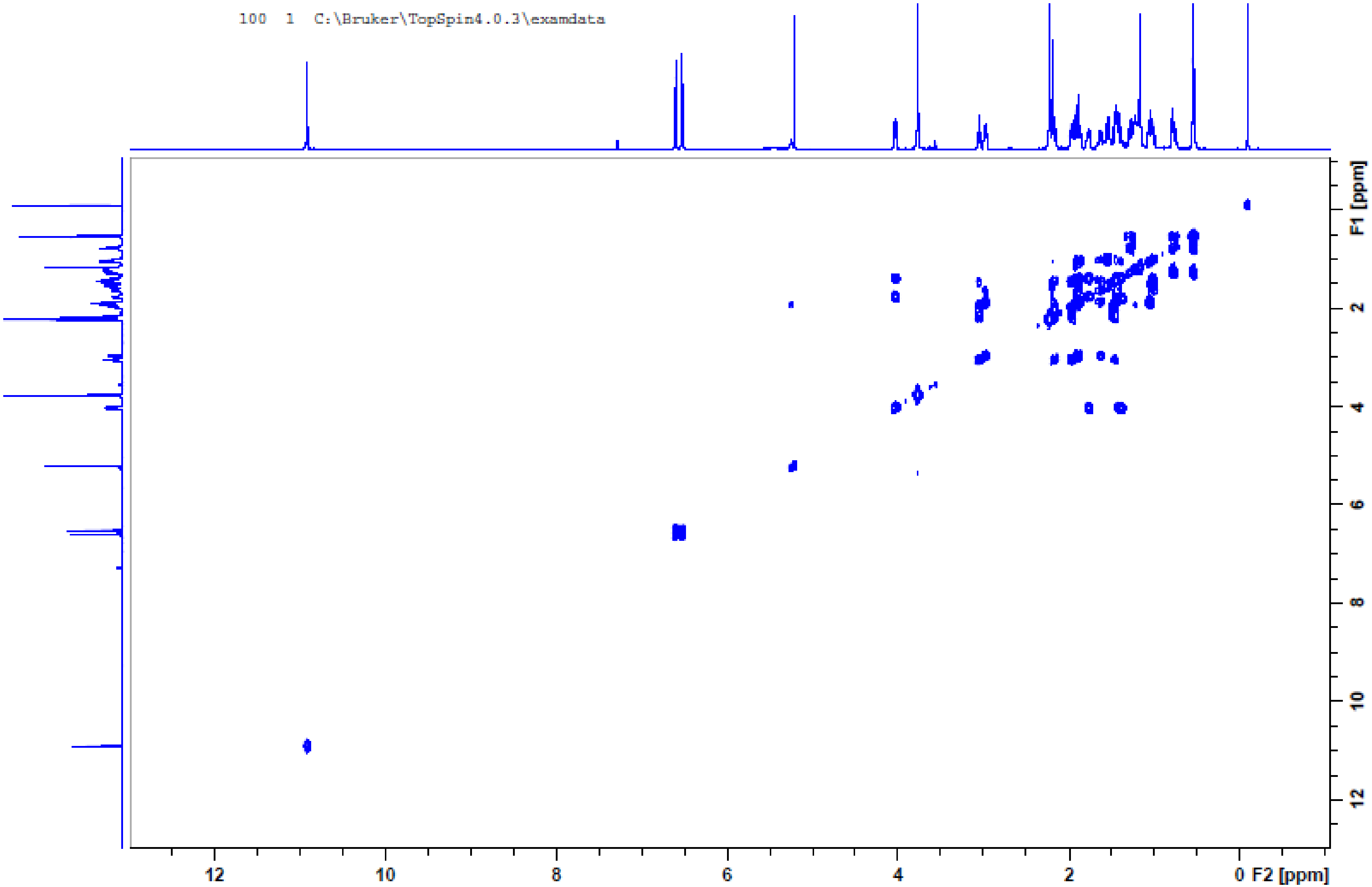

Figure S3: ^{1}H-^{1}1H-COSY spectrum of Aspidocarpine ($CDCl_3$, 500 MHz).

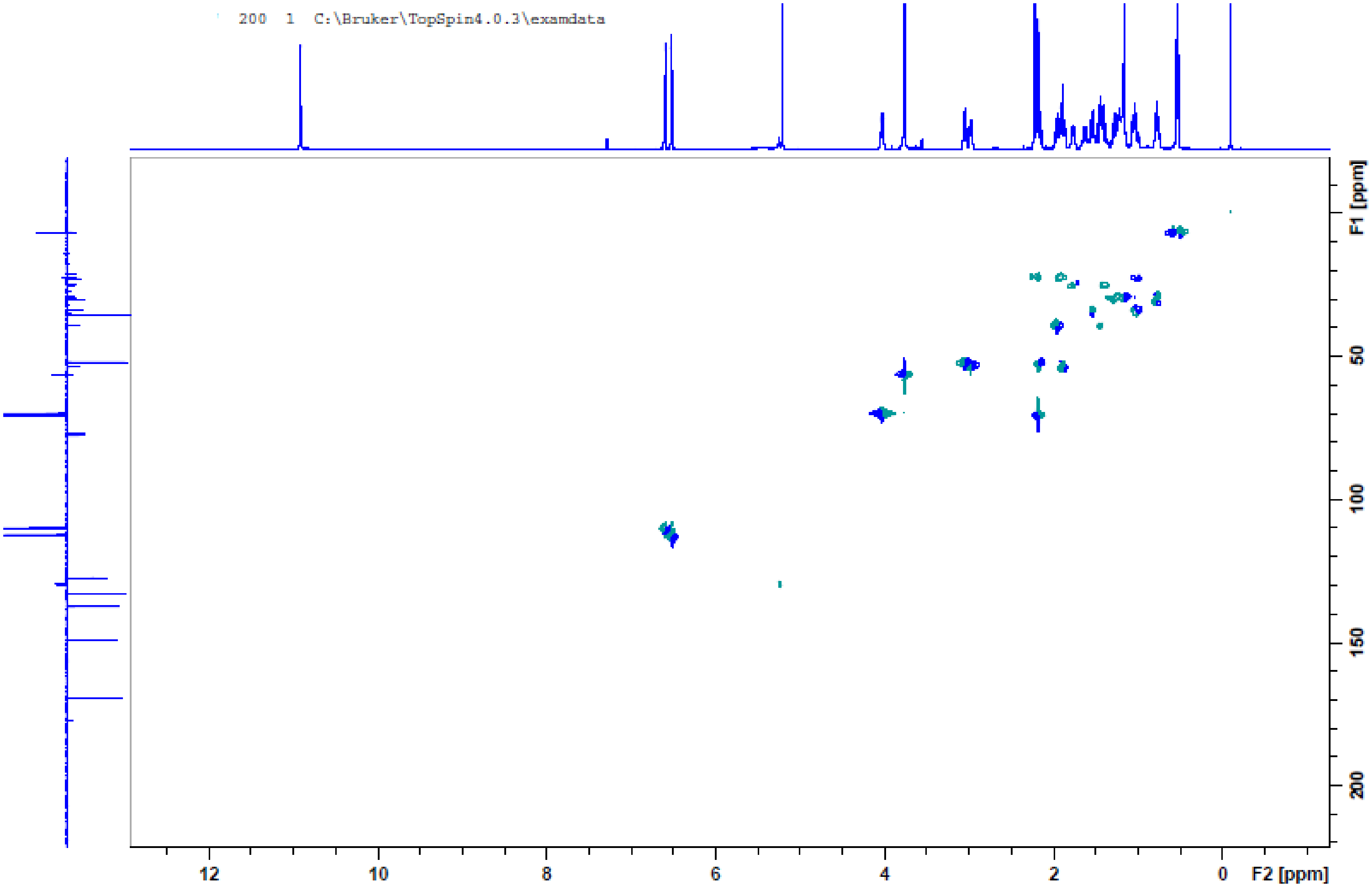

Figure S.4: HSQC spectrum of Aspidocarpine ($CDCl_3$, 500 MHz).

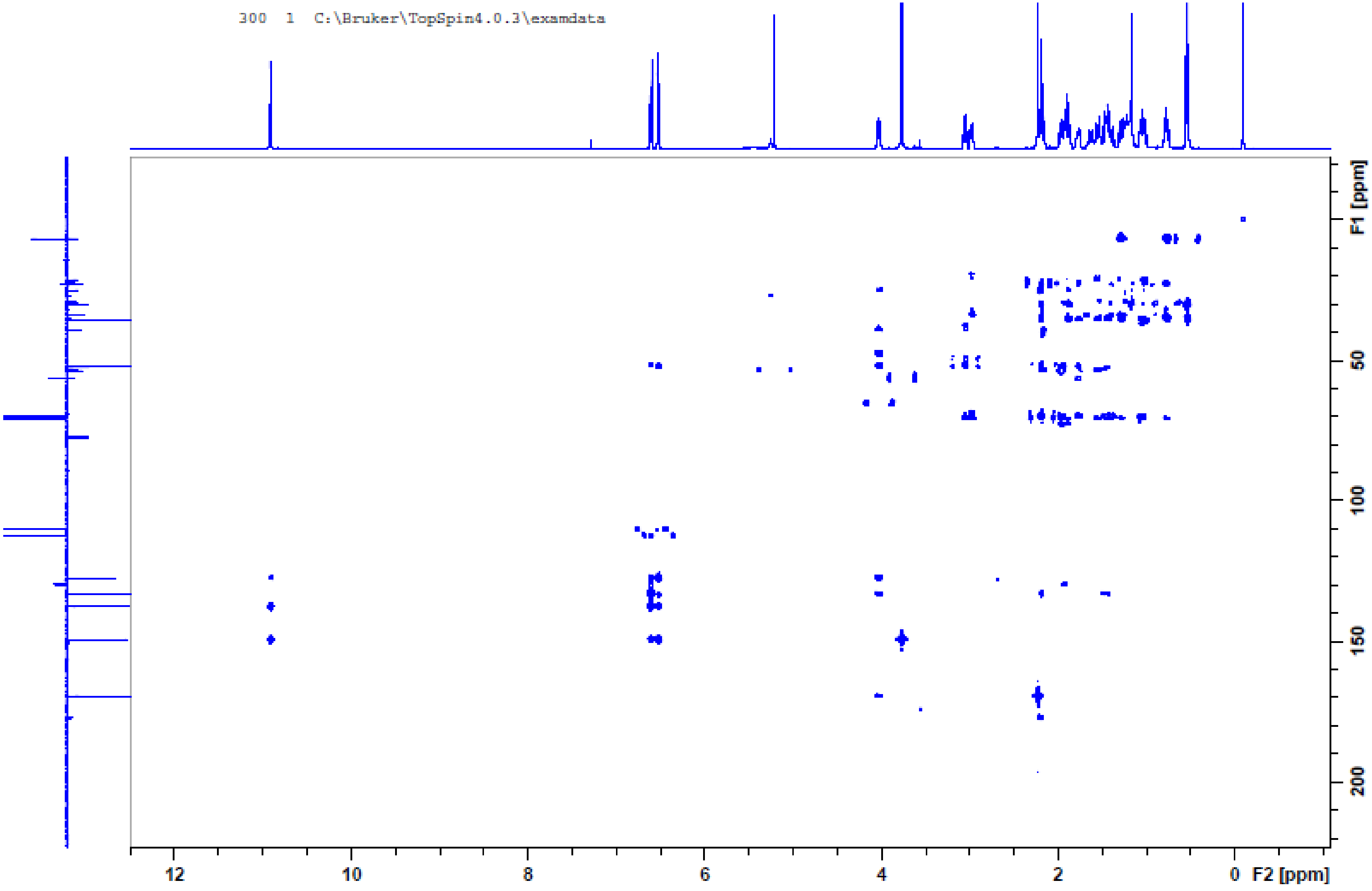

Figure S.5: HMBC spectrum of Aspidocarpine ($CDCl_3$, 500 MHz).

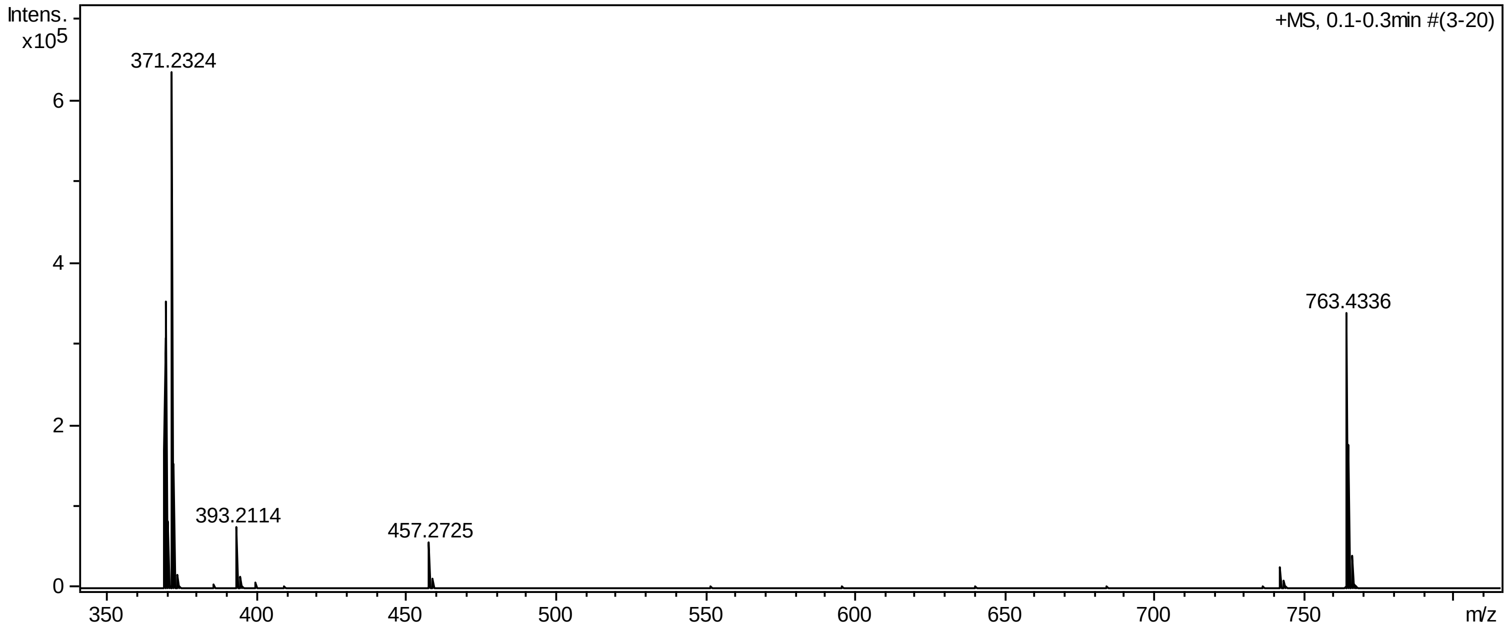

Figure S6: High-resolution mass spectrum (HRESIMS/positive mode) of Aspidocarpine.

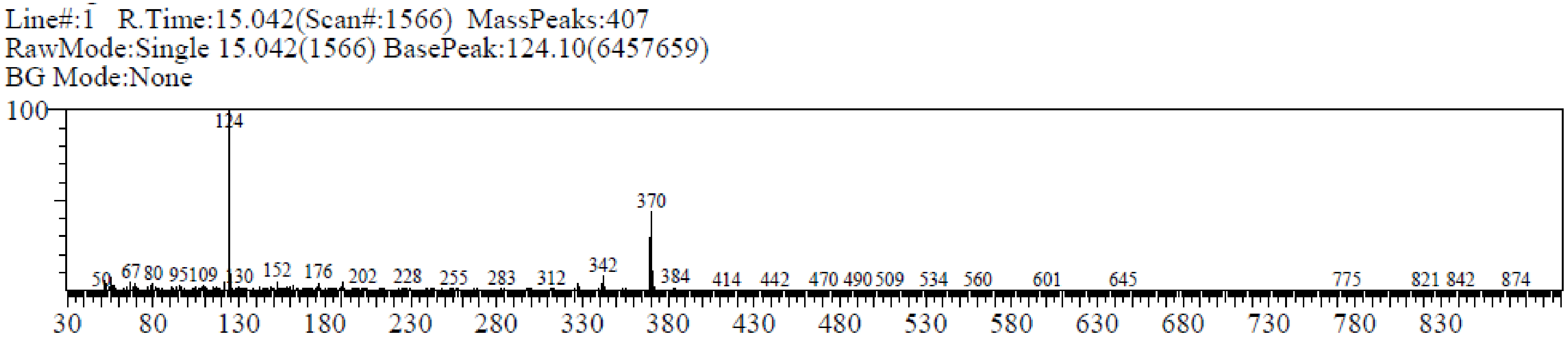

Figure S7: Low-resolution mass spectrum of Aspidocarpine.

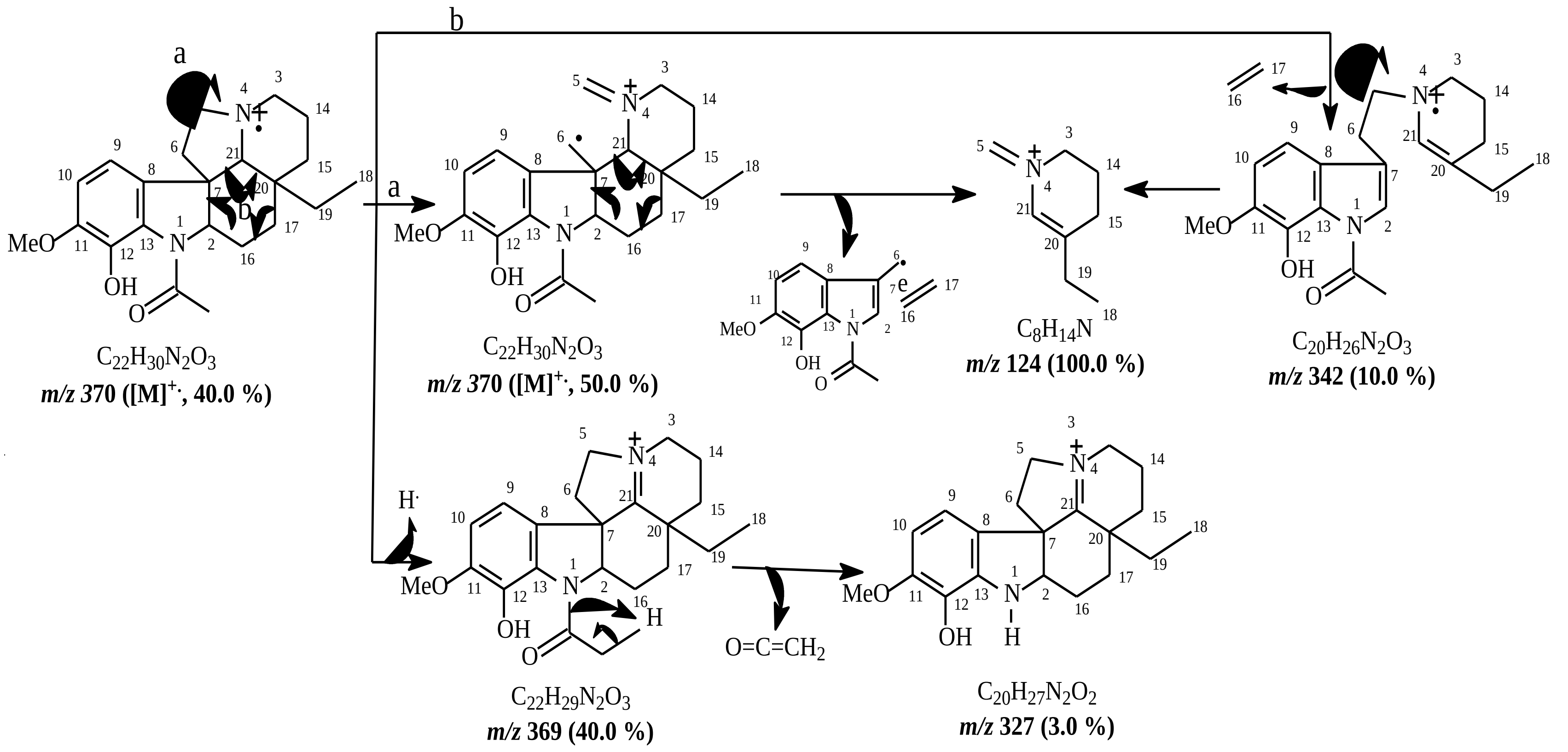

Figure S8: Proposal of mass spectra fragmentation of Aspidocarpine.

Table S1: ^{1}H (500 MHz) and ^{13}C (125 MHz) NMR spectral data for Aspidocarpine, including results obtained by heteronuclear 2D shift-correlated HSQC ($^{1}J_{CH}$) and HMBC ($^{n}J_{CH}$, n=2 and 3) and comparison with values described in the literature [16, 17]), in $CDCl_3$ as solvent. Chemical shifts (*d*, ppm) and coupling constants (*J*, Hz, in parenthesis).

	HMQC		HMBC		[17]		[16]	
	δ_C	δ_H	$^{2}J_{CH}$	$^{3}J_{CH}$	δ_C	δ_H	δ_C	δ_H
**C**								
7	51.99	-	H-2	2H-5; H-9	52.2	-	52.20	-
8	132.99	-	H-9	H-2; H-10; H-21	133.1	-	133.13	-
11	149.26	-	H-10	H-9; MeO-11; HO-12	149.4		149.35	-
12	137.36	-	HO-12	H-10	137.5		137.47	-
13	127.40	-		H-2; H-9; HO-12	127.5	-	127.51	-
20	35.42	-	2H-19	H-16a; 3H-18	35.5	-	35.46	-
1'	169.28	-	3H-2'	H-2	169.3	-	169.31	-
**CH**								
2	69.31	4.03 (dd, 11.3, 6.2)			70.3		70.25	4.07 (dd, 11.0, 6.0))
9	112.31	6.52 (d, 8.1)	H-10		112.4	6.61 (d, 8.0)	112.36	6.61 (d, 8.0)
10	109.99	6.60 (d, 8.1)	H-9		110.0	6.69 (d, 8.0)	110.04	6.69 (d, 8.0)
21	70.63	2.20 (s)		H-3a; H-5a; 2H-19	70.6	2.25 (s)	70.62	2.25 (s)
**CH_2**								
3	53.52	2.97 (dl, 10.9(<br>1.90			53.7	3.04 (dm, 12<br>1.98 (td, 12.0, 4.0)	53.67	3.04 (dm, 12.0) 1.98 (td, 12.0, 4.0)
5	52.27	3.06 (td, 9.0, 2.1)<br>2.15			52.4	3.12 (m),<br>2.27 (m)	52.43	3.12 (m),<br>2.27 (m)
6	39.16	1.98, 1.45	H-5b	H-2	39.4	2.04 (m), 1.57(m)	39.37	2.04 (m), 1.57 (m)
14	21.27	2.20, 1.92	H-15b		21.5	1.72(tm, 12.0)<br>1.53 (dm, 12.0, 4.0)	21.50	1.72 (tm, 12.0), 1.53 (dm, 12.0)
15	33.88	1.60, 1.02		H-3a	34.0	1.65 (dt, 12.0, 4.0)<br>1.11 (td, 12.0, 4.0)	34.02	1.65 (dt, 12.0, 4.0), 1.11 (dt, 12.0, 4.0)
16	24.99	1.88, 1.52			25.1	1.86 (m), 1.52 (m)	25.10	1.86 (m), 1.52 (m)
17	22.75	1.98, 1.20		H-19b	22.9	2.00 (td, 14.0, 12.0)<br>1.15 (dm, 12.0)	22.92	2.00 (td, 12.0, 14.0),<br>1.15 (dm, 12.0)
19	30.00	1.25, 0.80	3H-18		30.0	1.44 (m), 0.93 (m)	30.03	1.44 (m), 0.93 (m)
**CH_3**								
18	6.71	0.64 (t, 7.4)	2H-19		6.8	0.63 (t, 7.5)	6.76	0.63 (d, 7.5)
MeO	56.36	3.77 (s)			56.4	3.88 (s)	56.43	3.88 (s)
2'	22.60'	2,23 (s)			22.7	2.33 (s)	22,68	2.33 (s)
HO	-	10.91 (s)			-	10.98 (s)	-	10.98 (s)
